# Nationwide survey of late‐onset hemolysis in very low birthweight infants

**DOI:** 10.1111/ped.14493

**Published:** 2021-02-06

**Authors:** Yayoi Miyazono, Junichi Arai, Yu Kanai, Daisuke Hitaka, Daigo Kajikawa, Shusuke Takeuchi, Motomichi Nagafuji, Satoshi Fujiyama, Makoto Saito, Hidetoshi Takada

**Affiliations:** ^1^ Department of Child Health Faculty of Medicine University of Tsukuba Tsukuba Japan; ^2^ Department of Pediatrics University of Tsukuba Hospital Tsukuba Japan; ^3^ Department of Neonatology Ibaraki Children’s Hospital Mito Japan

**Keywords:** hemolytic anemia, hemolytic jaundice, kernicterus, very low birthweight infant, neonatal hyperbilirubinemia

## Abstract

**Background:**

In Japan, some cases of late‐onset acute hemolysis in very low birthweight (VLBW) infants have been reported. These cases had common features but the cause of hemolysis was unknown. The incidence and prognosis of this disease are also unknown. However, there are only few reports of such hemolytic episodes in countries other than Japan. Thus, this study aimed to examine the incidence and clinical course of late‐onset acute hemolysis and to establish it as a new disease concept.

**Methods:**

A nationwide prospective survey was conducted from 2011 to 2015 as a rare disease surveillance project of the Japan Society for Neonatal Health and Development.

**Results:**

Twenty‐four cases were confirmed. The median (range) gestational age, birthweight, and onset of hemolytic episodes were 26 weeks and 2 days (23 weeks and 4 days–31 weeks and 2 days), 898 g (627–1,416 g), and 19 days after birth (9–33 days), respectively. Phototherapy, blood transfusion, and exchange transfusion were required in 22 (96%), 24 (100%), and 7 (29%) cases, respectively. During the observation period, no recurrence of the hemolytic episode occurred. All patients survived; however, one case developed kernicterus and suffered severe neurological sequelae.

**Conclusions:**

In this study, at least 1 out of 1,259 VLBW infants developed hemolysis at 9–33 days after birth in Japan. Owing to the risk of kernicterus, this disease should be recognized as among the important pathological conditions of VLBW infants, suggesting the need to manage jaundice and anemia until 5 weeks after birth.

Kernicterus in premature infants is one of the important neonatal‐associated problems even in the present age when neonatal medicine has advanced in Japan.^1^ We previously reported four cases of acute hemolytic episodes of unknown cause in premature infants whose birthweights were <1,000 g at 2–4 weeks after birth.^2^ Since then, there have been some case reports with similar characteristics in Japan.^3^, ^4^, ^5^ These patients had some features in common, which are as follows: (i) all were very low birthweight (VLBW) infants who weighed <1,500 g at birth; (ii) episodes occurred suddenly at 10–30 days after birth; (iii) they had no family history of hemolytic diseases or blood type incompatibility between mother and baby; (iv) hemolysis was accompanied by an increase in methemoglobin (MetHb) concentration or the presence of Heinz bodies in erythrocytes, and (v) the hemolytic episodes were transient and not recurrent. There are few reports of such hemolytic episodes in countries other than Japan,^6^, ^7^, ^8^ but the incidence and prognosis of this disease in Japan are unknown.

We therefore aimed to investigate the clinical background, course, prognosis, and incidence of the relatively novel late‐onset acute hemolytic disease in VLBW infants by conducting a nationwide prospective survey in Japan.

## Methods

As a rare disease surveillance project of the Japan Society for Neonatal Health and Development (JSNHD), we conducted a nationwide prospective survey of acute hemolysis in VLBW infants after the acute phase from June 2011 to May 2015 for the academic members of the neonatologists working at the Japanese neonatal intensive care units.

To investigate the number of patients, primary questionnaires were sent along with the JSNHD journal, which was published three times a year to all academic members (approximately 3,000) during the study period. In the primary questionnaires, we asked them to contact us if they experienced a case of a hemolytic episode that met the following criteria: (i) VLBW infants (<1,500 g); (ii) the hemolytic episodes occurred after the acute phase (at 10–30 days after birth); (iii) the hemolysis was associated with the presence of Heinz bodies in red blood cells or increased MetHb concentration, and (iv) phototherapy, blood transfusion, or exchange transfusion was performed as treatment. Patients with blood‐type incompatibility between mother and baby, positive direct antiglobulin test, family history of congenital hemolytic diseases, or administration of drugs that induce methemoglobinemia, such as nitric oxide, were excluded. We then sent secondary questionnaires to the academic members who encountered the VLBW infants with hemolytic episodes to collect relevant information, including the detailed clinical course of both the mothers and patients. We investigated the profile of the infant, the history of the disease before the onset of hemolysis and its treatment, the age at which the hemolysis occurred, and the clinical course of hemolytic episodes. The prognosis was also examined. Informed consent was obtained from each parent, and the study was approved by the ethics committee of the University of Tsukuba.

### Statistical analyses

All statistical analyses were performed using IBM SPSS Statistics version 26.0 for Windows (IBM, Armonk, NY, USA). For the descriptive analysis, median, minimum, and maximum values were reported for continuous variables, and frequencies and percentages were reported for categorical variables.

## Results

### Primary survey

During the study period, 24 cases (male:female = 15:9) were confirmed from the 30 cases registered. Six cases were excluded for three reasons. First, the blood gas analyzer showed abnormal MetHb measurements such as a question mark or a minus sign instead of a numerical value in 3 cases. Second, nitric oxide was used in 2 cases. Third, neither MetHb concentration nor Heinz body was examined in one case. Although the hemolytic episodes in two cases occurred at 9 and 33 days after birth, these clinical courses were typical for this disease; thus, they were added to the confirmed cases.

### Secondary survey

The clinical data from 24 cases were collected and analyzed. The basic and perinatal data are presented in Table 1. The medians (ranges) of the birthweights and gestational ages were 898 g (627–1,416 g) and 26 weeks and 2 days (23 weeks and 4 days to 31 weeks and 2 days), respectively.

**Table 1:** Basic and perinatal data of 24 very low birthweight infants who developed late‐onset acute hemolysis from June 2011 to May 2015 in Japan

Basic data (*n* = 24)		
Gestational age (week), median (range)	26.3	(23.6–31.3)
Birthweight (g), median (range)	898	(627–1,416)
Apgar score (1 min), median (range)	5.5	(1–9)
Apgar score (5 min), median (range)	7	(4–9)
Male sex, *n* (%)	15	(63%)
Outborn,^†^ *n* (%)	1	(4%)
Cesarean section, n (%)	15	(63%)
Perinatal data (*n* = 24)		
Threatened premature delivery, *n* (%)	18	(75%)
Chorioamnionitis, *n* (%)	9	(38%)
Premature rupture, *n* (%)	9	(38%)
TTTS, *n* (%)	2	(8%)
Placental abruption, *n* (%)	1	(4%)
Placenta previa, *n* (%)	1	(4%)

^†^Outborn means an infant born in another hospital. TTTS, twin‐to‐twin transfusion syndrome.

The diseases and treatment before the onset of hemolysis are presented in Table 2. Because the diseases and treatments prior to the onset of hemolysis in these cases were common in general VLBW infants, we could not identify specific findings leading to the cause of the disease.

**Table 2:** Diseases and treatment before the onset of hemolysis

	*n*	(%)
Diseases before the onset of hemolysis (*n* = 24)		
Hyperbilirubinemia	24	(100)
RDS	19	(79)
Apnea of prematurity	11	(46)
PDA	8	(33)
IVH (grade Ⅰ or Ⅱ)	4	(17)
TTN	2	(8)
Treatment before the onset of hemolysis (*n* = 24)		
Phototherapy	24	(100)
Mechanical ventilation (MV)	22	(92)
MV at the onset of hemolysis	13	(54)
Oxygen	21	(88)
FIO_2_^ ^> 0.4	6	(25)
Surfactant	19	(79)
Antibiotics	19	(79)
Indomethacin	15	(63)
Xanthin	12	(50)
rhEPO	11	(46)
Iron preparation	10	(42)
γ‐Globulin	8	(33)
Blood transfusion	3	(13)

FIO_2_, fraction of inspiratory oxygen; IVH, intraventricular hemorrhage; PDA, patent ductus arteriosus; RDS, respiratory distress syndrome; rhEPO, recombinant human erythropoietin; TTN, transient tachypnea of the newborn.

Phototherapy was administered to all patients; the median (range) age during the start of phototherapy was 1 day (0–2 days), and the median duration of phototherapy was 3 days (1–9 days). The median of maximum total serum bilirubin (TSB) concentration before the onset of hemolysis was 7.5 mg/dL (4.9–12.6 mg/dL), and in 23 out of the 24 cases (95.8%), phototherapy was terminated before the onset of hemolysis. Three cases (12.5%) received blood transfusion.

Table 3 shows the details of the hemolytic episodes and treatment. The median age of hemolysis onset (range) was 19 (9–33) days after birth. In eight of the 24 cases (33％), Hb decreased to <7 g/dL, and the median of the minimum Hb level in each case was 7.5 g/dL (2.6–9.4 g/dL). The TSB increased to ˃15 mg/dL in 13 cases (54%), and the median of the maximum TSB concentration in each case was 15.0 mg/dL (3.3–24.3 mg/dL).

**Table 3:** Details of hemolytic episode

			*n*
Hemolytic episode			
Onset age (days after birth), median (range)	19	(9–33)	24
Minimum Hb (g/dL), median (range)	7.5	(2.6–9.4)	24
Maximum TSB (mg/dL), median (range)	15.0	(3.3–24.3)	23
Maximum USB (μg/dL), median (range)	1.28	(0.54–4.0)	8
Maximum MetHb (%), median (range)	3.0	(0.0–7.2)	20

	*n*	(%)
Heinz body (*n* = 24)		
Positive	18	(75)
Negative	2	(8)
Not examined	4	(17)
Complications (*n* = 24)		
Low SpO_2_	11	(46)
Tachycardia	1	(4)
Increased DSB	1	(4)
Treatment of hemolysis (*n* = 24)		
Phototherapy	23	(96)
Blood transfusion	24	(100)
Exchange transfusion	7	(29)
Others	7	(29)
Vitamin C	3	(13)
Albumin	2	(8)
γ‐globulin	2	(8)
Mechanical ventilation	1	(4)

DSB, direct serum bilirubin; Hb, hemoglobin; SpO_2_, peripheral capillary oxygen saturation; TSB, total serum bilirubin; USB, unbound serum bilirubin.

The maximum MetHb concentration was 3.0% (0.0%–7.2%), and Heinz bodies were present in 18 out of the 20 cases (90%), except for four untested cases. Complications at the onset of hemolysis were low SpO_2_ in 11 cases (45.8%), tachycardia in one case, and increase in direct bilirubin in one case.

In the treatment of hemolysis, 23 cases (96%) underwent phototherapy, and all cases received blood transfusion, including exchange transfusion in seven cases (29%).

To determine the cause of hemolysis, testing for congenital hemolytic disease was performed in four cases, of which one case underwent genetic testing (68 types) in addition to tests for erythrocyte enzyme activity, eosin‐5‐maleimide (EMA) binding ability, and isopropanol. No congenital hemolytic disease was identified.

Table 4 shows the prognosis of infants at 4–54 months of age (median 31 months). During the observation period, all patients survived, and there was no recurrence of a hemolytic episode. Nineteen cases (79%) had no sequelae, but one case of kernicterus (bilirubin encephalopathy) was accompanied by dyskinesia. At 11 months after birth, for this patient, abnormal high‐intensity areas on T2‐weighted magnetic resonance images were observed bilaterally in the globus pallidus.

**Table 4:** Prognosis during follow up at 4–54 months (median: 31 months)

	n	(%)
Survive	24	(100)
Recurrence of hemolytic episode	0	(0)
Sequelae		
Mild developmental delay	2	(8)
Kernicterus	1	(4)
Spastic cerebral palsy + bilateral hearing impairment	1	(4)
Motor development delay + unilateral hearing impairment	1	(4)

## Discussion

This is the first nationwide study on acute hemolysis with common features in VLBW infants. The present study suggests the necessity of recognizing the existence of late‐onset acute hemolytic episodes, which could cause severe jaundice and anemia after the acute period in VLBW infants. This disease can also cause severe neurological sequelae, such as kernicterus, so we named the disease “late‐onset hemolytic jaundice in VLBW infants.”

During the study period, 30 225 VLBW infants were born in Japan and survived ˃1 month after birth. At least one out of 1,259 VLBW infants developed sudden hemolysis after the acute phase in Japan.

Given that the criteria for the diagnosis of this disease include an increase in MetHb concentration or the appearance of Heinz bodies, it was considered that there were undiagnosed cases, where neither MetHb nor Heinz bodies were examined. Although cases with such clinical courses are rarely reported from other countries,^6^, ^7^, ^8^ they may be recognized if VLBW infants undergo these tests at 2–4 weeks after birth. No specific risk factors were found because the cases were in line with the usual treatment of VLBW infants and the present work was not a case‐control study.

The symptoms of the disease were similar to those previously reported in Japan.^2^, ^3^, ^4^, ^5^ The severity of the disease was variable but severe anemia with Hb <7 g/dL and jaundice with TSB concentration ˃15 mg/dL were observed in 33% and 54% of all cases, respectively. The proportion of cases requiring phototherapy and exchange transfusions was 96% and 29%, respectively, and all cases had received transfusions. It should be noted that this disease may cause severe jaundice. One case of kernicterus was strongly suspected to be associated with this hemolytic attack. This disease therefore needs to be diagnosed and treated appropriately. In this disease, it is necessary to note that for patients in whom jaundice occurs during the first few days of life, if they improve but then suddenly develop hemolysis, recognizing jaundice and anemia may be delayed. Thus, it is important to check the TSB, Hb, and MetHb levels frequently for the presence of Heinz bodies in erythrocytes during the first month of life. Nagasaka *et al*. reported that they could predict the onset of this disease using a transcutaneous bilirubin monitor, which was useful for the detection of the disease; thus, this device may be valuable for noninvasive TSB monitoring.^9^

As one of the accompanying symptoms, a decrease in SpO_2_ value was observed in 46% of cases. We have also experienced such a phenomenon in our past cases but no decrease in PaO_2_ level was observed despite the low SpO_2_. It was thought to be a false decrease due to the influence of MetHb.^10^ If this phenomenon is regarded as an acute exacerbation of chronic lung disease (CLD), increasing the fraction of inspiratory oxygen (FIO_2_) may adversely affect retinopathy of prematurity and CLD; thus, it is important to recognize this condition.

Given that Heinz bodies are inclusions within red blood cells composed of oxidized denatured hemoglobin,^11^ and MetHb results from oxidation of the iron moieties in the hemoglobin from the ferrous (Fe^2+^) to the ferric (Fe^3+^) state, oxidative stress in the erythrocytes of preterm infants was strongly suspected as a cause of hemolysis.^12^ In fact, some reports have suggested that erythrocytes of neonates, particularly premature infants, are vulnerable to oxidative stress because of the imbalance between the production of oxidant species and activities of antioxidants.^13^, ^14^, ^15^, ^16^, ^17^ We also previously reported that erythrocyte enzyme activities in extremely low birthweight infants have different characteristics from those of adults.^18^ In the report, we showed that erythrocyte glucose‐6‐phosphate dehydrogenase (G6PD) activity was higher in preterm infants than in adults, whereas 6‐phosphogluconate dehydrogenase (6PGD) activity was significantly lower, resulting in a markedly decreased 6PGD/G6PD ratio. It was suggested that the reduced 6PGD/G6PD ratio in very preterm infants would lead to an increased susceptibility to oxidative stress in combination with markedly decreased activity of glutathione peroxidase, methemoglobin reductase, and catalase, which protect erythrocytes from oxidative damage. However, it is unclear why the hemolytic episodes occur only in certain VLBW cases. The existence of a specifically Japanese genetic background is also suspected because there are few case reports of hemolytic episodes with common characteristics similar to this disease reported from countries other than Japan.

Generally, hemolytic anemia with Heinz bodies includes hereditary hemolytic diseases, such as G6PD deficiency and hemoglobinopathies. Four of the 24 cases were searched for hereditary hemolytic diseases, such as hereditary spherocytosis, G6PD deficiency, or unstable hemoglobin in this study, but no cases were diagnosed with the disease. In addition, extrinsic factors such as exposure to phenol or naphthalene are known to cause Heinz body hemolytic anemia,^6^, ^19^ and administration of methylene blue to the fetus and newborn infant,^20^, ^21^ or use of lidocaine‐prilocaine cream^22^ for the newborn is known to cause MetHb elevation, but these were not used in the patients in this study.

There are several limitations to our study. First, as this study was dependent on voluntary case registration from neonatal physicians nationwide, it was not an all‐case study; thus, the actual number of occurrences was estimated to be higher. An all‐case study will be required in order to investigate the actual number of late‐onset acute hemolysis occurrences. Second, there might be a few cases that have not been diagnosed with this disease, where neither Heinz bodies nor MetHb was examined. By widely recognizing the existence of this disease, early detection and treatment of hemolysis are expected, with frequent monitoring of the TSB, Hb, and MetHb levels and Heinz bodies in VLBW. Furthermore, only four of the 24 cases were examined for congenital hemolytic diseases. In the previous case reports of premature infants with G6PD deficiency, the onset of hemolysis occurs within the first week of life in most male infants but our case does not match the clinical course previously reported.^23^, ^24^, ^25^, ^26^, ^27^ In addition, G6PD deficiency is a rare disease in patients with Japanese ethnicity,^28^ but the possibility of hereditary hemolytic disease, such as G6PD deficiency, cannot be completely ruled out. Thus, it is also necessary to examine the possibility of a hereditary hemolytic disease, such as G6PD deficiency, in future studies.

Moreover, further investigation is needed to elucidate the cause and etiology of late‐onset hemolytic jaundice in VLBW infants in future studies. Future research will need to evaluate the imbalance between the degree of oxidative stress and antioxidants in VLBW infants with and without hemolytic episodes as well as their genetic background.

### Conclusion

We have established a new disease concept of late‐onset hemolytic jaundice in VLBW infants through a nationwide survey. In Japan, at least one out of 1,259 VLBW infants suddenly developed hemolysis at 9–33 days after birth. Given that this disease can cause kernicterus, it is important to recognize its presence. To improve the clinical outcome and prognosis in VLBW infants, it is necessary to monitor the TSB, Hb, and MetHb levels of infants frequently during the first month of life for the early detection and treatment of hemolysis.

## Funding information

The authors have not received any specific grant for this study from any funding agency in the public, commercial, or not‐for‐profit sectors.

## Disclosure

The authors declare no conflict of interest.

## Author contributions

All authors met the criteria of ICMJE. M.Y. designed the study and wrote the initial draft of the manuscript. A.J. and K.Y. performed the analysis and assisted in the preparation of the manuscript. T.H. revised the manuscript critically for important intellectual content. All other authors have contributed to data collection and interpretation and critically reviewed the manuscript. All authors contributed to the intellectual content of this manuscript and approved the final manuscript as submitted.
